# Comparison of Different Blood Transfusion Methods in Patients Undergoing Cesarean Section

**DOI:** 10.3389/fsurg.2022.844984

**Published:** 2022-02-22

**Authors:** Fei Guo, Heshan Tang, Xiaoqiang Wei

**Affiliations:** Department of Blood Transfusion, The First Affiliated Hospital of Naval Military Medical University, Shanghai, China

**Keywords:** cesarean section, allogeneic transfusion, acute normovolemic hemodilution, autologous transfusion, application effect

## Abstract

**Purpose:**

To compare the effect of allogeneic transfusion and acute normovolemic hemodilution (ANH) autologous transfusion in patients undergoing cesarean section.

**Methods:**

Patients who underwent cesarean section and received blood transfusion therapy from February 2019 to July 2021 in our hospital were observed and divided into the allogeneic group (n = 55) who received allogeneic transfusion therapy and the autologous group (n = 55) who received ANH autologous transfusion therapy according to the mode of transfusion. Observations included vital signs [heart rate (HR), mean arterial pressure (MAP), stroke volume variation (SVV)], blood routine [red blood cells (RBC), platelets (PLT), hematocrit (HCT), hemoglobin (Hb)], T-cell subsets (CD4^+^, CD8^+^, CD4^+^/CD8^+^), immunoglobulins (IgA, IgM, IgG), inflammatory factors [C-reactive protein (CRP), tumor necrosis factor (TNF)-α, interleukin (IL)-6], and adverse effects were counted in both groups.

**Results:**

There was no statistical significance in the intra-group and inter-group comparisons of HR, MAP, and SVV between the two groups before transfusion and transfusion for 10 min (*P* > 0.05). 5d after operation, the RBC, PLT, HCT, and Hb of the allogeneic group were lower than those before operation, and the autologous group was higher than that of the allogeneic group (*P* < 0.05). 5d after operation, the CRP, TNF-α, and IL-6 of the allogeneic group were higher than those before operation, and the autologous group was lower than that of the allogeneic group (*P* < 0.05). 5d after operation, the CD4^+^, CD4^+^/CD8^+^ of the allogeneic group were lower than before operation, and the CD8^+^ was higher than before operation. The CD4^+^ and CD4^+^/CD8^+^ of the autologous group were higher than that of the allogeneic group, and CD8^+^ was lower than that of the allogeneic group (*P* < 0.05). 5d after operation, the IgA, IgG, and IgM of the allogeneic group were lower than those before operation, and the autologous group was higher than that of the allogeneic group (*P* < 0.05). During blood transfusion, there was no significant difference in the adverse reaction rate between the two groups (*P* > 0.05).

**Conclusion:**

Both allogeneic transfusion and ANH autologous transfusion have little effect on the vital signs of patients undergoing cesarean section, but ANH autologous transfusion is more helpful to the stability of blood routine, T-cell subsets, immunoglobulin, and inflammation levels after surgery, which is a safe and effective way of blood transfusion.

## Introduction

Cesarean section is an important midwifery procedure in the field of obstetrics. It is suitable for cases where the fetus cannot be delivered from the vagina normally, such as cephalopelvic error, birth canal abnormalities, fetal distress, fetal position error, umbilical cord prolapse, history of cesarean section, multiple births, etc. (1, 2). Placenta praevia and placental abruption are common complications of cesarean section, which can exacerbate the incidence of perinatal hemorrhage in cesarean section, and the incidence is much higher than that of natural delivery, therefore, in obstetric surgery, the blood transfusion rate of cesarean section 0.77% is higher than natural delivery 0.23% (3, 4). Preoperative blood preparation is an important preoperative preparation for cesarean section. It can effectively reduce the risk of disseminated intravascular coagulation (DIC), shock and even death in patients with cesarean section bleeding. Therefore, it is beneficial to improve the uterine retention rate and survival rate of pregnant women. For patients with large blood loss during cesarean section, urgent blood transfusion is often required in clinic, currently, allogeneic blood transfusion is mainly used, but it is associated with postoperative infection, immunosuppression and a poor prognosis (5). Acute normovolemic hemodilutio (ANH) autologous transfusion is an autologous blood transfusion method in which autologous blood is drawn preoperatively and supplemented with an equal volume of crystal or colloidal fluid, and the patient's blood loss is combined during the operation to return the autologous blood (6).

The acute normovolemic hemodilutio (ANH) autologous transfusion is a form of autotransfusion in which autologous blood is drawn preoperatively and replenished with an equal volume of crystalloid or colloidal fluid, and then returned autologous blood intraoperatively according to the amount of blood lost by the patient (6). It can effectively reduce the hematocrit (HCT), reduce the loss of blood elements during bleeding, improve the body's tolerance after hemodilution, and shorten the time of ischemia and hypoxia in patients through blood dilution, which is a blood conservation technique that can reduce the risk of anesthesia and surgery and provide fresh whole blood to the patient (7). It has been widely used in major surgeries such as orthopedics, oncology and neurosurgery, but there are very few applications and related reports in the field of obstetrics (8, 9). This study compares the application effect of allogeneic transfusion and ANH autologous transfusion in patients undergoing cesarean section, aiming to explore the effectiveness and safety of ANH autologous transfusion in patients undergoing cesarean section.

## Materials and Methods

### Research Object

Patients who underwent cesarean section and received blood transfusion from February 2019 to July 2021 in our hospital were used as observation subjects. Inclusion criteria: age 20–35 years old; proposed cesarean section; American Society of Anesthesiologists (ASA) classification II–III (10); normal liver and kidney function, normal cardiopulmonary function; normal four items before transfusion; normal coagulation function; preoperative platelet (PLT) > 100 × 10^9^/L, HCT > 33%, hemoglobin (Hb) > 110 g/L (11); patients or her family had signed the informed consent. Exclusion criteria: people undergoing allogeneic and autologous transfusion at the same time; people who had a history of heart disease or tumor disease; people who had a history of neurological or psychiatric disease; people with immune system diseases; people with systemic acute and chronic infections; People with cognitive and communication impairments. They were divided into the allogeneic group (*n* = 55) receiving allogeneic transfusion therapy and the autologous group (*n* = 55) receiving ANH autologous transfusion therapy according to the mode of blood transfusion. There was no significant difference between the two groups in terms of age, gestational weeks and other general conditions, which were comparable, which were comparable (*P* > 0.05) (Table 1).

**Table 1:** Comparison of patients' general conditions (*n, M* ± *SD*, %).

**Indexs**	**Allogeneic group (*n* = 55)**	**Autologous group (*n* = 55)**	***t*/χ^2^ value**	***P* value**
Age (years old)	28.02 ± 3.11	28.76 ± 2.98	1.274	0.205
Gestational week (weeks)	36.54 ± 0.59	36.62 ± 0.61	0.699	0.486
Body mass index (kg/cm^2^)	26.85 ± 3.24	27.03 ± 3.26	0.290	0.772
Operative time (h)	1.57 ± 0.83	1.61 ± 0.79	0.259	0.796
Blood loss (mL)	953.25 ± 26.01	956.24 ± 25.47	0.609	0.544
Number of outputs (times)			0.147	0.702
1	24 (43.64)	26 (47.27)		
≥2	31 (56.36)	29 (52.73)		
History of cesarean section (cases)			0.042	0.838
No	37 (67.27)	38 (69.09)		
Yes	18 (32.73)	17 (30.91)		
ASA classification (cases)			0.146	0.702
II	28 (50.91)	30 (54.55)		
III	27 (49.09)	25 (45.45)		
Maternity status (cases)			0.326	0.955
Placenta previa	38 (69.09)	39 (70.91)		
Placental abruption	5 (9.09)	4 (7.27)		
Placental implantation	3 (5.46)	4 (7.27)		
Others	9 (16.36)	8 (14.55)		

### Research Methods

Patients in both groups underwent cesarean section and general anesthesia was induced intraoperatively. On this basis, the allogeneic group received allogeneic transfusion therapy, i.e., when HCT <24% or Hb <80 g/L, stock blood was taken for transfusion perfusion according to the amount of blood lost by the patient. The autologous group received ANH autologous transfusion therapy, i.e., radial artery and right internal jugular vein puncture placement were performed after induction of anesthesia and before the start of surgery. Preoperatively, according to the patient's intraoperative bleeding prediction, the CZK-IB microcomputer liquid sampling controller (purchased from Zhengzhou Feilong Medical Equipment Co., Ltd.) was used to collect 300–420 mL of autologous blood through the radial artery and stored in a blood storage bag and treated with light shielding and freshness. An equal volume of 6% hydroxyethyl starch (HES) 130/0.4 (purchased from Shandong Hualu Pharmaceutical Co., Ltd., approval number H37022757) was then infused *via* the internal jugular vein. autologous blood was transfused at the end of the main intraoperative step or when the bleeding volume was ≥ 1,000 mL or Hb was ≤ 100 g/L.

### Observation Index

(1) Vital signs: heart rate (HR), mean arterial pressure (MAP), and stroke volume variation (SVV) were monitored before transfusion and transfusion for 10 min by a PICCO monitor (purchased from Beijing Shimao Medical Equipment Trading Co., Ltd.).

(2) Blood routine: The red blood cell (RBC), PLT, HCT and Hb levels were measured before and 5 d after surgery by MAXM automatic hematology analyzer (purchased from Beckman Coulter, Inc.).

(3) T-cell subsets: The CD4^+^, CD8^+^, CD4^+^/CD8^+^ levels were measured before and 5 d after surgery by a Cytomics FC500 flow cytometer (purchased from Beckman Coulter, Inc.).

(4) Immunoglobulins: The IgA, IgM, and IgG levels were measured before and 5 d after surgery by enzyme-linked immunosorbent assay (The kit was purchased from Roche).

(5) Inflammatory factors: C-reactive protein (CRP), tumor necrosis factor (TNF)-α and interleukin (IL)-6 levels were measured before and 5 d after surgery by enzyme-linked immunosorbent assay (The kit was purchased from Shanghai Sange Biotechnology Co., Ltd.).

(6) Adverse reaction rate: Allergy, fever, hemolysis and other adverse reactions occurred during blood transfusion in the two groups were counted.

### Statistical Methods

SPSS 22.0 software was applied, and the measurement data were expressed as mean ± standard deviation (*M* ± *SD*) and compared by *t*-test. Count data were expressed as ratio, and the χ^2^ test was used for comparison. *P* < 0.05 was considered statistically significant.

## Results

### Effect of Different Blood Transfusion Methods on Patients' Vital Signs

**Figure 1:**
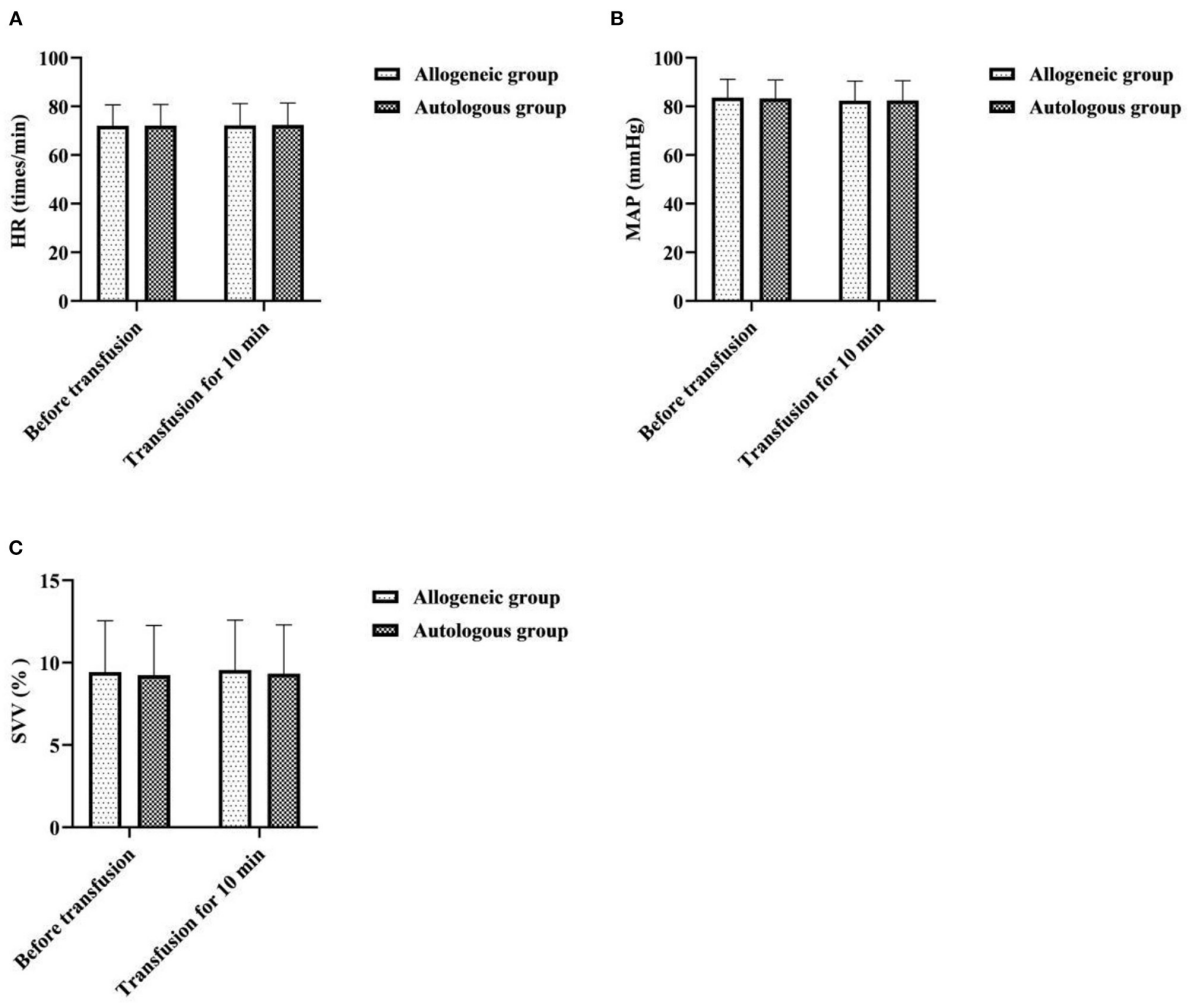
Effect of different blood transfusion methods on patients' vital signs. **(A)** HR, **(B)** MAP, and **(C)** SVV.

There was no statistical significance in the intra-group and inter-group comparisons of HR, MAP, and SVV between the two groups before transfusion and transfusion for 10 min (*P* > 0.05) (Figure 1).

### Effect of Different Blood Transfusion Methods on Patients' Blood Routine

**Figure 2:**
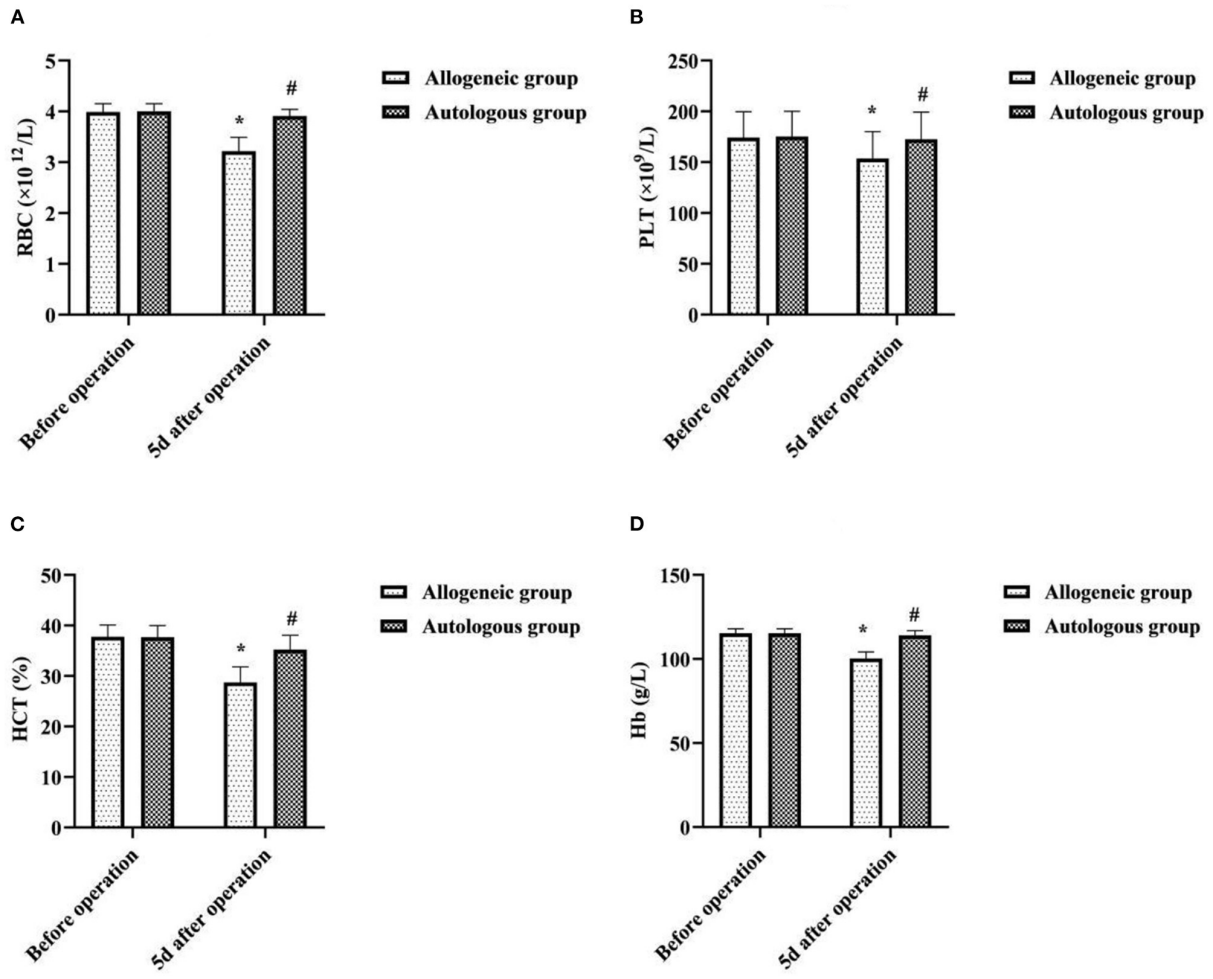
Effect of different blood transfusion methods on patients' blood routine. **(A)** RBC, **(B)** PLT, **(C)** HCT, and **(D)** Hb. Compared with the same group before operation, *P < 0.05; compared with allogeneic group 5d after operation, ^#^*P* < 0.05.

5d after operation, the RBC, PLT, HCT, and Hb of the allogeneic group were lower than those before operation, and the autologous group was higher than that of the allogeneic group (*P* < 0.05) (Figure 2).

### Effect of Different Blood Transfusion Methods on Patients' Inflammatory Factors

**Figure 3:**
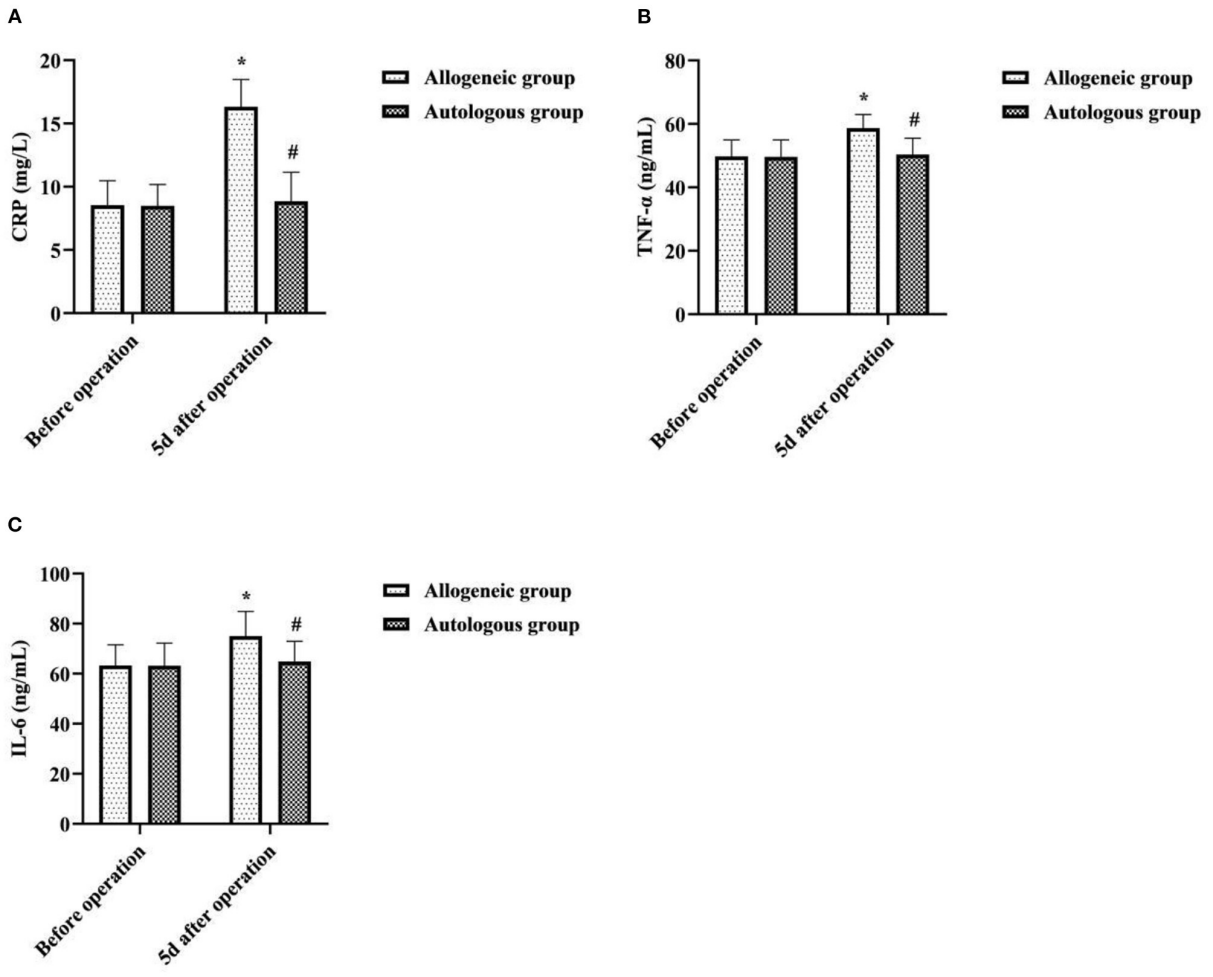
Effect of different blood transfusion methods on patients' inflammatory factors. **(A)** CRP, **(B) TNF-α**, and **(C)** IL-6. Compared with the same group before operation, *P < 0.05; compared with allogeneic group 5d after operation, ^#^*P* < 0.05.

5d after operation, the CRP, TNF-α, and IL-6 of the allogeneic group were higher than those before operation, and the autologous group was lower than that of the allogeneic group (*P* < 0.05) (Figure 3).

### Effect of Different Blood Transfusion Methods on Patients' T-Cell Subsets

**Figure 4:**
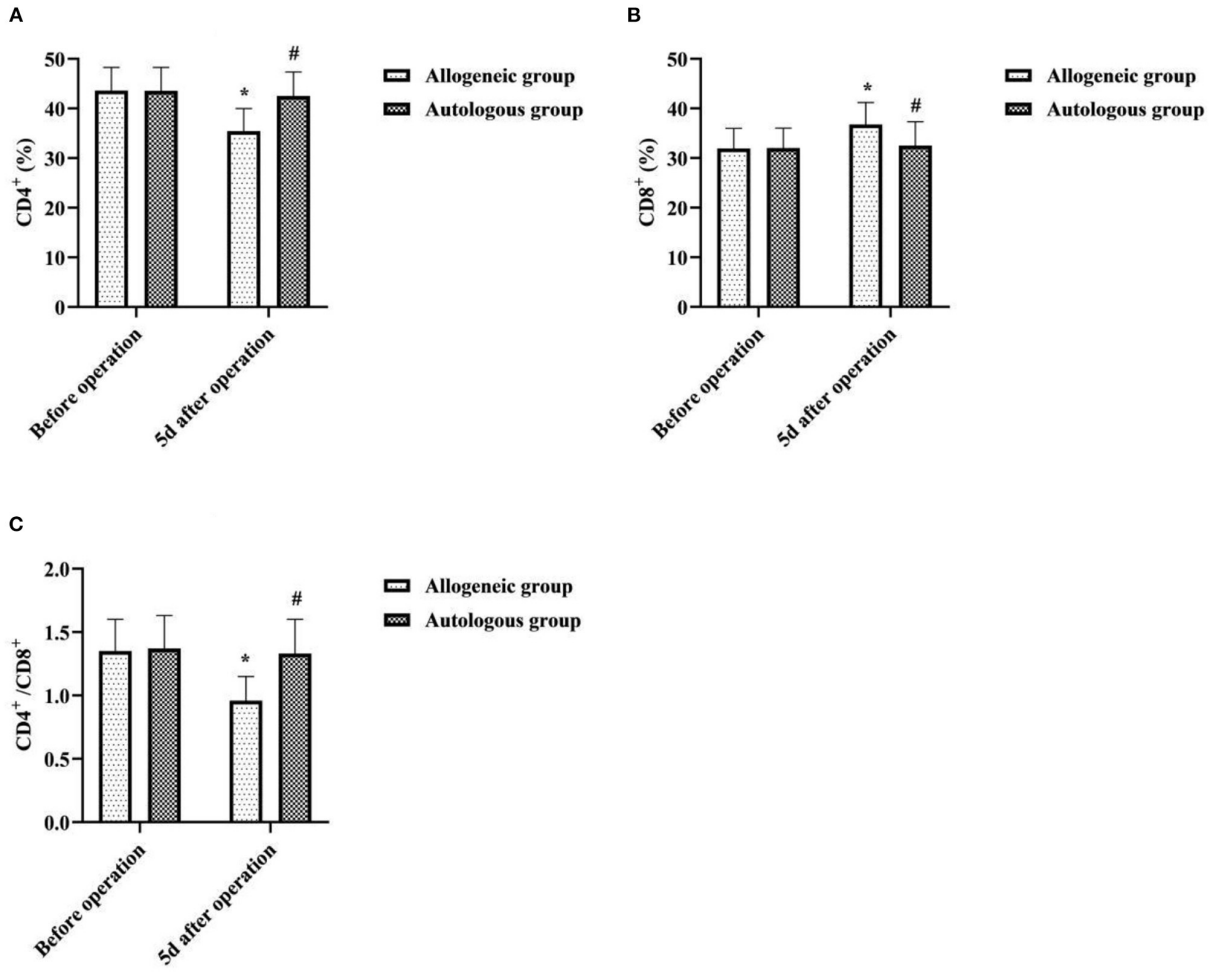
Effect of different blood transfusion methods on patients' T-cell subsets. **(A)** CD4^+^, **(B)** CD8^+^, and **(C)** CD4^+^/CD8^+^. Compared with the same group before operation, *P < 0.05; compared with allogeneic group 5d after operation, ^#^*P* < 0.05.

5d after operation, the CD4^+^, CD4^+^/CD8^+^ of the allogeneic group were lower than before operation, and the CD8^+^ was higher than before operation. The CD4^+^ and CD4^+^/CD8^+^ of the autologous group were higher than that of the allogeneic group, and CD8^+^ was lower than that of the allogeneic group (*P* < 0.05) (Figure 4).

### Effect of Different Blood Transfusion Methods on Patients' Immunoglobulins

**Figure 5:**
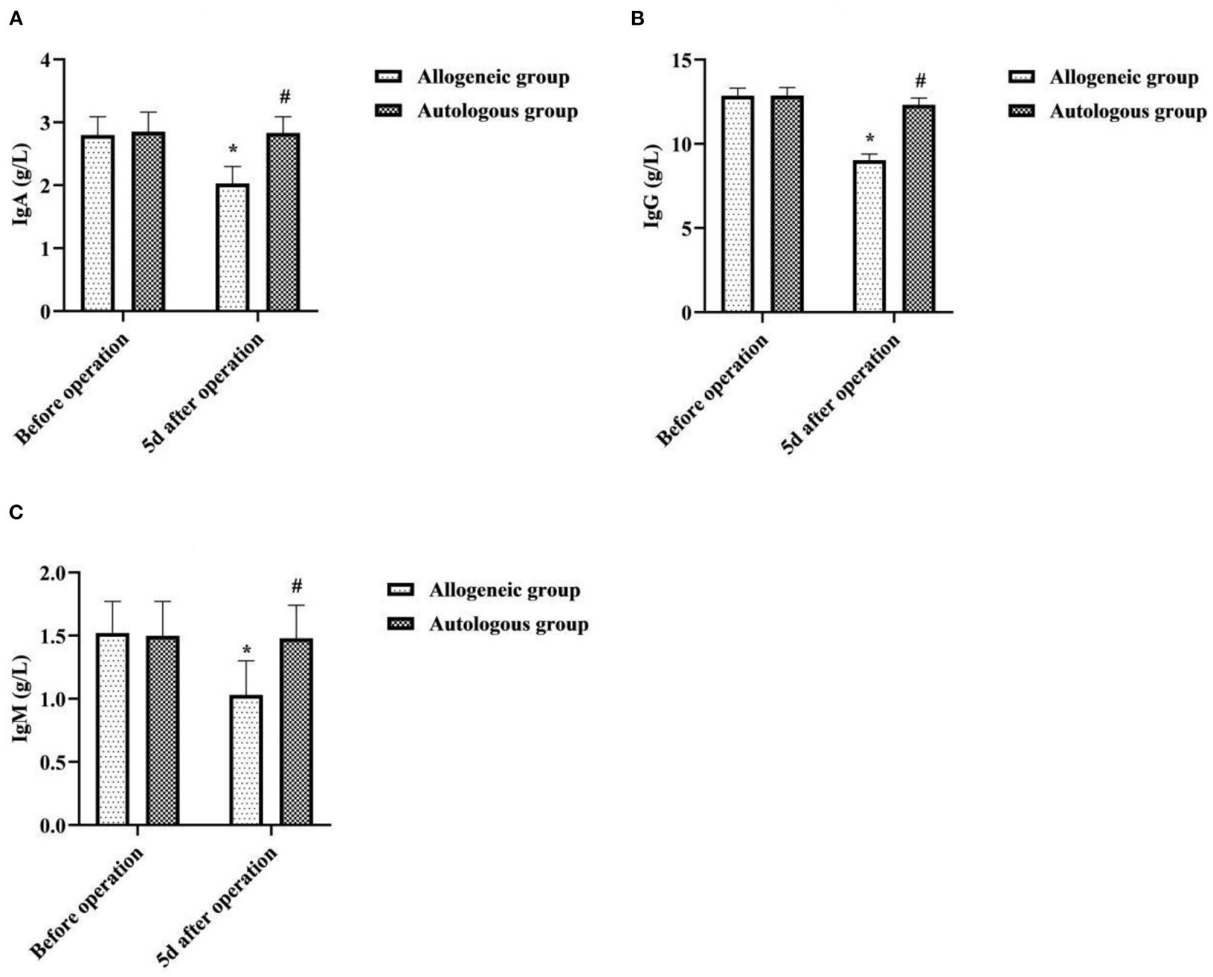
Effect of different blood transfusion methods on patients' immunoglobulin's. **(A)** IgA, **(B)** IgG, and **(C)** IgM. Compared with the same group before operation, *P < 0.05; compared with allogeneic group 5d after operation, ^#^*P* < 0.05.

5d after operation, the IgA, IgG, and IgM of the allogeneic group were lower than those before operation, and the autologous group was higher than that of the allogeneic group (*P* < 0.05) (Figure 5).

### Adverse Reaction Rate of Different Blood Transfusion Methods

During blood transfusion, there was no significant difference in the adverse reaction rate between the two groups (*P* > 0.05) (Table 2).

**Table 2:** Adverse reaction rate of different blood transfusion methods (*n*, %).

**Group**	**Allergy**	**Fever**	**Hemolysis**	**Others**	**Total**
Allogeneic group (*n* = 55)	1 (1.82)	2 (3.64)	1 (1.82)	3 (5.45)	7 (12.73)
Autosomal group (*n* = 55)	1 (1.82)	1 (1.82)	0 (0.00)	2 (3.64)	4 (7.28)
*χ^2^* value					0.909
*P* value					0.340

## Discussion

In recent years, due to social factors such as late marriage and late childbirth, and second child policy, the number of advanced maternal age and scarred uterus re-pregnancy in China has been increasing, and the cesarean delivery rate has also increased, and in some areas it has exceeded 50% (12). It is reported that cesarean section patients are prone to severe bleeding that is not easy to control during the perinatal period, which is one of the main reasons for the poor prognosis or even death of mothers and babies (13). So, timely, reasonable, and adequate blood transfusion treatment is of great significance for suppressing perinatal hemorrhage and ensuring the safety of mothers and babies. Allogeneic transfusion is the main method at present, but it has been plagued by problems such as tight blood source and many adverse reactions of blood transfusion. It has been reported that ANH autologous transfusion may be superior to allogeneic blood transfusion in terms of blood safety (14). However, this blood transfusion method uses exogenous fluid for dilution, and whether the blood transfusion will affect the vital signs, immune function, and inflammation levels of patients undergoing cesarean section is still unknown. This study provided a comparative analysis in this regard.

It has been suggested that blood volume increases in women after pregnancy and that ANH autologous transfusion can achieve a reduction in blood viscosity, increase blood oxygen uptake, reduce cardiac burden, and protect the myocardium through preoperative blood sampling and dilution (15). In this study, there was no statistical significance in the intra-group and inter-group comparisons of HR, MAP, and SVV between the two groups before transfusion and transfusion for 10 min (*P* > 0.05). It showed that ANH autologous transfusion helps to maintain hemodynamic stability in patients undergoing cesarean section. To analyze the reasons, HES, as an artificial colloidal fluid with large molecular weight, has the pharmacological properties of being unable to penetrate the vessel wall, long intravascular retention time, and good effect of maintaining plasma colloidal osmotic pressure (16). Therefore, compared with isotonic crystalloids, it can achieve hemodynamic stability with less dosage and faster effect, and is one of the most commonly used resuscitation fluids in hemorrhagic shock. Surgery may result in loss of tangible components of blood, and allogeneic transfusions may result in destruction of blood components due to the long storage time of blood. In this study, 5d after operation, the RBC, PLT, HCT, and Hb of the allogeneic group were lower than those before operation, and the autologous group was higher than that of the allogeneic group (*P* < 0.05). It was suggested that ANH autologous transfusion is an effective way to improve hematoprotection and prevent the development of postoperative anemia. Analyze the reasons. Compared with allogeneic blood transfusion, ANH autologous blood transfusion is performed by drawing autologous blood before surgery and returning it to the patient during surgery, which not only reduces the loss of red blood cells and platelets during surgery, but also has a short storage time and does not require refrigeration, so the blood components are less damaged, which facilitates the patient's postoperative recovery. CRP, TNF-α, and IL-6 are all key cytokines that initiate inflammatory or immune responses when the body perceives inflammatory stimuli such as trauma (17, 18). In this study, 5d after operation, the CRP, TNF-α, and IL-6 of the allogeneic group were higher than those before operation, and the autologous group was lower than that of the allogeneic group (*P* < 0.05). It was suggested that allogeneic transfusion can lead to varying degrees of inflammatory response in patients undergoing cesarean section, whereas ANH autologous transfusion has a mild effect on the level of inflammation in patients. This may be related to the fact that the blood dilution of ANH autotransfusion reduces concentrations of cortisol and catecholamines in plasma, and that the blood released out of the body after hemodilution is not involved in the acute phase response.

As an immunogenic and reactogenic substance, blood can be accompanied by a series of adverse reactions involving immune regulation in the process of blood transfusion therapy, mainly manifested as immunosuppression (19). Both T-cell subsets and immunoglobulins are important indicators to assess the immune function of the body, with the former playing a central regulatory role in cellular immunity and the latter being closely related to humoral immunity undertaken by B cells (20, 21). When allogeneic blood enters the human body as foreign protein antigen, the differentiation of T lymphocytes into CD4^+^ cells is inhibited, cytotoxic T lymphocytes (CD8^+^ T cells) are activated, and then the proportion of CD4^+^/CD8^+^ is unbalanced, resulting in abnormal immune function. In this study, 5d after operation, the CD4^+^, CD4^+^/CD8^+^, IgA, IgG and IgM of the allogeneic group were lower than before operation, and the CD8^+^ was higher than before operation. The CD4^+^, CD4^+^/CD8^+^, IgA, IgG and IgM of the autologous group were higher than that of the allogeneic group, and CD8^+^ was lower than that of the allogeneic group (*P* < 0.05). It indicated that allogeneic transfusion can cause a decrease in immune function in the recipient, while ANH autologous transfusion has less effect on immune function in patients undergoing cesarean section. Analysis of the causes may be related to a decrease in the immune function of red blood cells due to the long storage time of the stock blood used for allogeneic transfusion. Numerous studies (22–24) have shown that the erythrocyte system has some immune functions that cannot be replaced by other immune cells, namely, reducing free radical damage, scavenging immune complexes, and participating in immune defense. Under normal circumstances, stock blood used for allogeneic transfusion can be stored at a constant temperature of 4°C for 2–3 weeks. However, the longer the storage time, the more serious the deformation and aging of RBCs, the gradual decrease of RBC-C3b receptor activity, the excessive accumulation of related metabolites, the increase of immune complexes, and finally the impaired immune function of RBCs (25). In contrast, autologous blood of ANH autologous transfusion has a short retention time outside the body and does not require refrigeration, has few changes in red blood cells and their associated metabolites, and is free of alloantigens and proteins, and has few white blood cell fragments, thus causing minimal suppression of the immune system. The results of this study also showed that the incidence of adverse transfusion reactions such as allergy, fever, and hemolysis was slightly lower in the autologous group than in the allogeneic group (*P* > 0.05). It can be seen that ANH autologous transfusion is safer and will not increase the incidence of adverse blood transfusion reactions.

## Conclusion

Through the comparative analysis of the above results, we found that both allogeneic and ANH autologous transfusion had little effect on the vital signs of patients undergoing cesarean section, but ANH autologous transfusion was more helpful in stabilizing the postoperative blood routine, *T*-cell subpopulation, immunoglobulin, and inflammation levels, and was a safe and effective way of blood transfusion. It is worth noting that although ANH autologous transfusion is a safe and effective way of blood transfusion, it can not completely replace allogeneic blood transfusion. Some studies (26) pointed out that when high-dose HES is used, due to the dilution effect, it may cause dose-related abnormal blood coagulation and decrease of HCT. Therefore, when the body has massive bleeding and the amount of recovered blood is also large, RBC, PLT and coagulation factors need to be supplemented at the same time in order to avoid serious coagulation dysfunction. The specific blood transfusion method should also depend on the specific situation of the patient.

## Data Availability Statement

The original contributions presented in the study are included in the article/supplementary material, further inquiries can be directed to the corresponding author/s.

## Ethics Statement

The studies involving human participants were reviewed and approved by the Ethics Committee of the First Affiliated Hospital of Naval Military Medical University. The patients/participants provided their written informed consent to participate in this study.

## Author Contributions

FG and HT are responsible for the design of the study and manuscript writing. XW is the instructor of the entire study, responsible for the inclusion of cases, and data statistics. All authors contributed to the article and approved the submitted version.

## Conflict of Interest

The authors declare that the research was conducted in the absence of any commercial or financial relationships that could be construed as a potential conflict of interest.

## Publisher's Note

All claims expressed in this article are solely those of the authors and do not necessarily represent those of their affiliated organizations, or those of the publisher, the editors and the reviewers. Any product that may be evaluated in this article, or claim that may be made by its manufacturer, is not guaranteed or endorsed by the publisher.
